# Safety of Fecal Microbiota Transplantation in Children: A Single-Center Experience

**DOI:** 10.3390/children13081005

**Published:** 2026-07-29

**Authors:** Dominykas Varnas, Vaidotas Urbonas

**Affiliations:** Clinic of Children’s Diseases, Institute of Clinical Medicine, Faculty of Medicine, Vilnius University, 03101 Vilnius, Lithuania

**Keywords:** fecal microbiota transplantation, pediatric, colonoscopy, safety, adverse events

## Abstract

**Highlights:**

**What are the main findings?**

- In a single-centre retrospective cohort of 73 children undergoing 108 colonoscopy-delivered FMT procedures, the short-term adverse event rate was 10.2%, with all events graded CTCAE 1–2 and no serious adverse events.
- The most common adverse events were diarrhoea (5.6%) and vomiting (2.8%); adverse event rates varied by underlying indication, with the lowest rate observed in procedures performed for autism spectrum disorder.

**What are the implications of the main findings?**

- Colonoscopy-delivered FMT in children appears safe in the short term, supporting its continued use for recurrent *C. difficile* infection and its careful investigation in other emerging pediatric indications.
- Underlying disease activity (e.g., recurrent CDI, IBD) may influence procedural risk and should be taken into account in the design of future studies.

**Abstract:**

**Background/Objectives:** Fecal microbiota transplantation (FMT) is an established treatment for recurrent or refractory *Clostridioides difficile* infection (rCDI) and is increasingly investigated for other gastrointestinal and neuropsychiatric conditions in children. However, pediatric safety data remains limited. This study aimed to evaluate the short-term safety of colonoscopy-delivered FMT in a heterogeneous pediatric cohort. **Methods:** We conducted a retrospective single-centre cohort study of all FMT procedures performed at Vilnius University Hospital Santaros Klinikos between 2017 and 2025. Fecal material from three screened healthy pediatric donors was delivered to the caecum via colonoscopy under general anesthesia. Adverse events (AEs) arising within 72 h of each procedure were identified from the clinical records and assigned a severity grade using version 5.0 of the Common Terminology Criteria for Adverse Events (CTCAE). AEs were analyzed on a per-procedure basis. Associations between sex, age, and underlying indication and AE occurrence were assessed using Fisher’s exact test and the Mann–Whitney U test. **Results:** A total of 108 FMT procedures were performed on 73 children aged 2 to 17 years. The predominant indication was autism spectrum disorder (ASD) and other developmental disorders (80.6%). AEs were reported after 11 procedures (10.2%); all were classified as CTCAE grade 1 or 2, and no serious AEs occurred. The most common AEs were diarrhoea (5.6%) and vomiting (2.8%). In an exploratory comparison, procedures performed for ASD were associated with a lower AE rate compared with other indications combined (6.9% vs. 23.8%; OR 0.24, 95% CI 0.06–0.87). **Conclusions:** Colonoscopy-delivered FMT was well tolerated in pediatric patients, with a favorable short-term safety profile consistent with published pediatric FMT literature. As follow-up was largely passive and the cohort was predominantly children with ASD, the observed rate should be regarded as a lower bound, and these findings warrant confirmation in prospective studies.

## 1. Introduction

The human gut is home to a diverse and continually changing community of microorganisms that contributes to host metabolism, shaping of the immune system, and maintenance of the intestinal lining. Quantitative, qualitative or functional disruptions to microbial balance, called dysbiosis, have been associated with intestinal as well as systemic diseases [1,2,3]. As a result, there is growing interest in developing strategies to modulate the gastrointestinal microbial ecosystem to prevent or treat various pathological conditions. Among these, fecal microbiota transplantation (FMT) has been emerging as a direct and effective method to improve dysbiosis and restore a healthy gut microbial ecosystem [4,5]. FMT involves introducing a minimally processed microbial community and metabolites, prepared from the stool of a healthy donor, into the gut of a recipient in order to alter that recipient’s own microbiota [6]. With FMT, the recipient receives not only microorganisms but also microbial metabolites and food residues, which may have both beneficial and adverse effects. The first FMT in children was reported in 2010 [7]. Currently, FMT is considered as a standard treatment for recurrent or refractory *Clostridioides difficile* infection (rCDI) and it is the only indication confirmed by North American Society for Pediatric Gastroenterology, Hepatology, and Nutrition (NASPGHAN) and European Society for Pediatric Gastroenterology, Hepatology, and Nutrition (ESPGHAN) consensus guidelines [8]. Outside CDI, FMT is being explored across a range of other conditions: gastrointestinal ones including inflammatory bowel disease (IBD) [9], irritable bowel syndrome (IBS) [10], and constipation [11], and neuropsychiatric ones such as autism spectrum disorder (ASD) [12], Parkinson’s disease [13], and anorexia nervosa [14,15]. For all of these conditions other than rCDI, FMT remains investigational. Although the therapeutic benefits of FMT in CDI are well recognised, and being researched in other conditions, the data regarding the adverse events (AE) associated with the procedure especially in the children population is limited [16,17]. The adverse effects reported most often are mild and gastrointestinal, including nausea, vomiting, diarrhoea, abdominal pain, flatulence, and low-grade fever [18].

**Figure 1:**
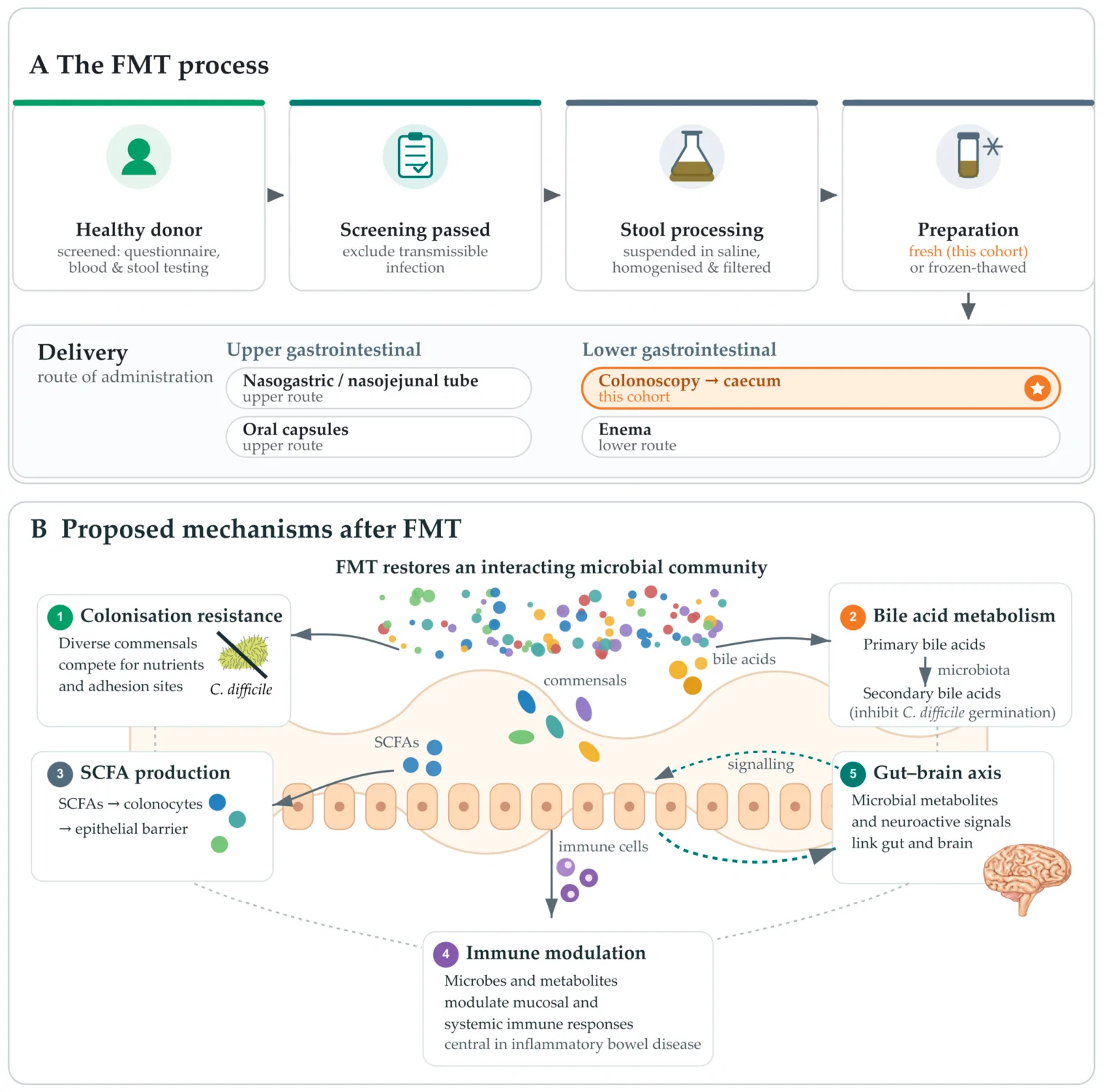
Overview of faecal microbiota transplantation (FMT): procedure and proposed mechanisms of action. (**A**) The FMT process. Stool is obtained from a healthy donor who has passed questionnaire, blood and stool screening to exclude transmissible infection; the sample is suspended in saline, homogenised and filtered, and administered either fresh (as in the present cohort) or after freeze–thawing. FMT may be delivered by the upper gastrointestinal route (nasogastric/nasojejunal tube or oral capsules) or the lower gastrointestinal route (colonoscopic delivery to the caecum, as used in this cohort, or enema). (**B**) Proposed mechanisms through which FMT restores an interacting microbial community: (1) colonisation resistance, in which diverse commensals compete with pathogens for nutrients and adhesion sites and lower luminal pH or produce inhibitory compounds; (2) bile-acid metabolism, in which the restored microbiota convert primary bile acids to secondary bile acids that inhibit *C. difficile* germination and outgrowth; (3) short-chain fatty acid (SCFA) production, which nourishes colonocytes and supports the epithelial barrier; (4) immune modulation, in which microbes and their metabolites modulate mucosal and systemic immune responses, of particular relevance in inflammatory bowel disease; and (5) gut–brain axis signalling, in which microbial metabolites and neuroactive signals link the gut and brain.

The rationale for FMT is based on restoring a depleted or imbalanced microbial community to a more diverse and functional state, increasingly understood as the re-establishment of cooperative microbial networks rather than the simple replacement of missing species [19], although the underlying mechanisms are only partly understood and differ between indications [20]. A varied microbiota restores colonisation resistance, whereby resident commensals hold pathogens in check by outcompeting them for nutrients and attachment sites, acidifying the lumen, and releasing inhibitory substances [21]. In recurrent *C. difficile* infection this effect is reinforced once normal bile-acid handling is re-established, since a healthy microbial community transforms primary bile acids into secondary forms that suppress *C. difficile* spore germination and growth [22]. Fermentation by gut microbes additionally generates short-chain fatty acids, which fuel colonocytes, support the integrity of the epithelial barrier, and help regulate mucosal immunity [23,24,25]. Beyond the intestine, microbial metabolites and neuroactive signals are believed to operate along the gut–brain axis, offering a rationale for exploring FMT in neuropsychiatric conditions such as ASD [26,27] (Figure 1).

In practice, FMT proceeds through several steps (Figure 1). A healthy donor is selected and screened with a structured questionnaire and laboratory testing of blood and stool to exclude potentially transmissible infection. Donor stool is suspended usually in saline, homogenised, and strained to remove solid matter, and the resulting preparation may be administered fresh or after freezing and thawing. Delivery may be via the upper gastrointestinal tract (nasogastric or nasojejunal tube, or oral capsules) or the lower tract (colonoscopy or enema), with bowel preparation generally preceding lower-route administration [8]. However, important uncertainties remain regarding how FMT is best performed: from the route of delivery, donor screening, and stool preparation to the conditions it should treat and the strength of the underlying safety and efficacy evidence [28]. The donor screening, stool processing, and delivery protocol used in the present cohort are detailed in the Materials and Methods.

Adverse events and their frequency differ among these delivery routes, which makes route-specific safety data even scarcer. Given the paucity of safety data in children, the present study aims to conduct a retrospective cohort analysis focusing specifically on the colonoscopy method.

## 2. Materials and Methods

### 2.1. Study Population

FMT was performed as individualized clinical care under a pre-existing hospital clinical protocol in place at our centre since 2017, operating independently of any research activity. The indication for FMT in each child was determined on a case-by-case basis by a multidisciplinary clinical consilium according to the individual clinical situation. For non-established indications, including ASD, the consilium met with the parents or legal guardians before the procedure and explicitly disclosed that, under current guidelines, FMT is not a standard or approved treatment for these conditions. FMT was offered as an additional option and was never a precondition for access to standard care. Parents and legal guardians were not pressured to choose FMT; the decision was entirely voluntary, and declining FMT did not affect the child’s access to standard care or any other services. Standard care for children with ASD at our institution consists of individualized multidisciplinary non-pharmacological interventions (kinesitherapy, speech and language therapy, occupational therapy, structured teaching, and art therapy), determined and continued at the discretion of the treating clinicians independently of FMT.

Clinical informed consent for the procedure was obtained from the parents or legal guardians before each FMT as part of routine clinical care. Separately, for the present retrospective analysis, use of the patients’ medical records was approved by Vilnius Regional Bioethics Committee (Approval No.: 4-1400-893, approved 12 April 2022), and the study was conducted in accordance with the Declaration of Helsinki. The committee’s approval covered the retrospective collection and analysis of clinical data rather than the procedures themselves.

**Figure 2:**
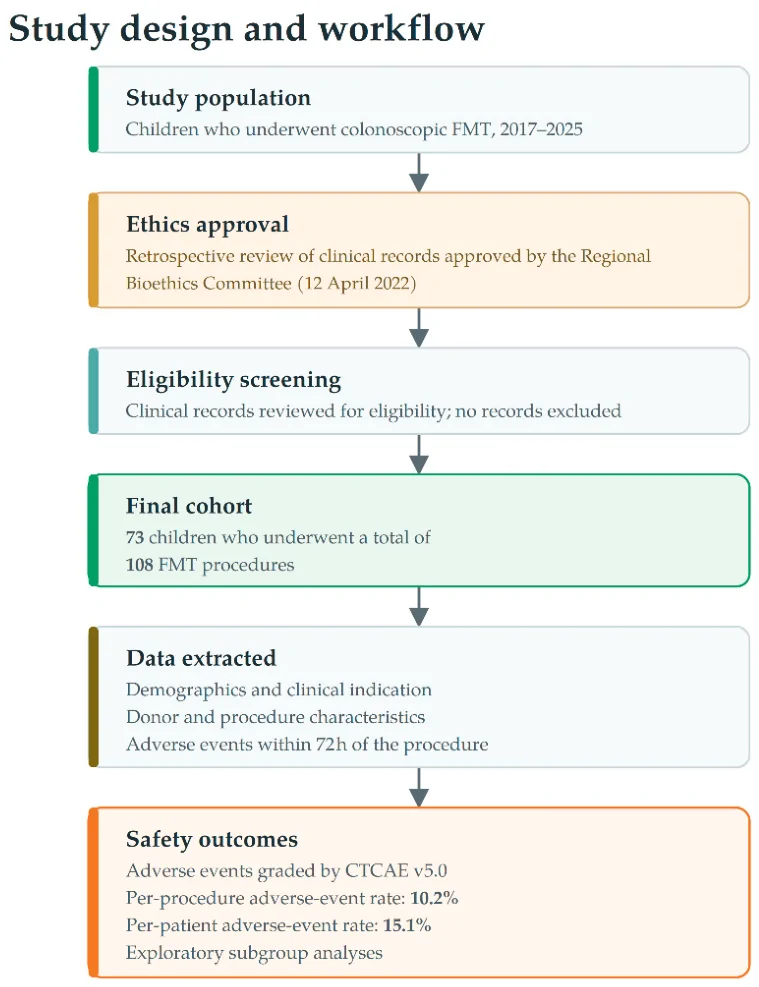
Overview of the study design and workflow. CTCAE, Common Terminology Criteria for Adverse Events; FMT, faecal microbiota transplantation.

An overview of the study design and workflow is provided in Figure 2.

### 2.2. Choice of Donors, FMT Preparation and Procedure

#### 2.2.1. Donor Eligibility and Exclusion Criteria

Stool was donated by three unrelated healthy children aged 3–7 years, who participated as volunteers without any payment or compensation. Candidate donors were evaluated through a structured medical interview and had to meet the following requirements:

Eligibility required:
  - Good general health, with no symptoms at the time of donation;
  - A regular bowel pattern and well-formed stools.
A candidate was not accepted if any of the following applied:
  - A known food allergy;
  - Intake of antibiotics, probiotics, or other medication in the preceding 30 days;
  - A chronic illness in the donor or their close family.

Comprehensive donor screening investigations were repeated every three months. In addition, a short health questionnaire was completed before each individual stool donation to identify any interim changes in health status, recent medication use, gastrointestinal symptoms, or potential infectious exposures. Only donors with negative results on all screening tests and no concerning findings on the pre-donation questionnaire were eligible to participate.

During the COVID-19 pandemic period, SARS-CoV-2 RT-PCR testing from nasopharyngeal swabs was additionally performed on donors prior to donation; 26 of the 108 procedures in this cohort were performed during the pandemic period in Lithuania (February 2020–May 2022).

#### 2.2.2. Stool Screening Panel

Each donor stool sample was tested across several categories before use. Parasitological assessment comprised microscopy for protozoa and helminth ova, alongside a fecal occult blood test. Adenovirus, norovirus, and rotavirus were sought on viral testing. A PCR-based bacterial panel covered *Campylobacter* spp., *Salmonella* spp., *Shigella* spp., *Vibrio* spp., *Yersinia* spp., toxin-producing *Escherichia coli*, *Listeria monocytogenes*, vancomycin-resistant *Enterococcus* (VRE), and methicillin-resistant *Staphylococcus aureus* (MRSA); *Clostridioides difficile* toxins A and B were also assayed.

#### 2.2.3. Blood Screening Panel

Venous blood was also drawn from every donor to rule out transmissible infection. The serological work-up covered hepatitis A virus IgM, hepatitis B surface antigen (HBsAg), hepatitis C virus antibodies (anti-HCV), human immunodeficiency virus (HIV), cytomegalovirus (CMV) IgM, and Epstein–Barr virus (EBV) IgM, with syphilis assessed by the rapid plasma reagin (RPR) test. A full blood count, C-reactive protein (CRP), and erythrocyte sedimentation rate (ESR) were obtained in addition.

#### 2.2.4. FMT Preparation

Fresh stool was used for all procedures. Donor stool samples were collected and stored at +4 °C, and in all cases the interval between collection and FMT was less than 8 h. Stool was diluted in 0.9% NaCl at an approximate 1:1 ratio (approximately 1 g of stool per 1 mL of saline); absolute amounts ranged from 50 to 300 g of stool in 50 to 300 mL of saline, scaled to the recipient’s age and weight. The suspension was homogenized using a blender and filtered to remove undigested material and solid particles. After preparation, the fecal suspension was administered within 1 h.

#### 2.2.5. Bowel Preparation and FMT Procedure

Patients underwent bowel preparation with 1.5–3 L of polyethylene glycol 4000 (Fortrans, Beaufour Ipsen Industrie, Dreux, France) or a weight-based dose of sodium picosulfate with magnesium citrate (Picoprep, Ferring GmbH, Kiel, Germany) solution, administered the day before and on the morning of the procedure. No routine pre-FMT antibiotic decolonization regimen was used. Pre-FMT antibiotic exposure differed by indication: two patients in the rCDI group received oral vancomycin as part of their clinical care, in both cases completed more than five days before FMT. Patients treated for other indications did not receive antibiotics before FMT.

The fecal solution was delivered to the caecum via colonoscopy under general anesthesia with sevoflurane administered by mask. The whole FMT procedure lasted from 10 to 20 min.

### 2.3. Evaluation of FMT-Related Adverse Events

Only short-term AE were analyzed in this study. Short-term AE were defined as any medical occurrence within 72 h of the FMT procedure, including new symptoms as well as exacerbations of pre-existing conditions. As this was a retrospective study, AE data were extracted from inpatient medical records and nursing documentation.

Follow-up combined active in-hospital monitoring with passive post-discharge contact. The duration of in-hospital observation was determined primarily by logistical factors, including parental preference and distance from the hospital; most patients remained hospitalized for at least 24 h after the procedure, while a subset was discharged on the day of the procedure. Patients who developed AE during the in-hospital observation period were monitored for a longer duration as clinically indicated. Throughout the inpatient stay, symptoms, vital signs, and clinical status were systematically documented. Upon discharge, all patients and their parents or legal guardians were provided with direct contact information of the gastroenterologist who performed the procedure and were instructed to report any new or worsening symptoms occurring within the 72 h follow-up window.

No scheduled follow-up contact was performed; AE assessment was considered complete at the end of the inpatient stay unless the family contacted the team. Information obtained through post-discharge contacts was recorded in the patient’s medical record and included in the analysis if it occurred within 72 h of the procedure.

The severity of each AE was classified using version 5.0 of the Common Terminology Criteria for Adverse Events (CTCAE) [29]. AEs were analyzed on a per-procedure basis rather than per-patient. Given the retrospective design and the heterogeneity of underlying conditions, indications for repeat FMT, and intervals between procedures, the per-procedure approach allows a more granular assessment of procedural safety and avoids underestimating cumulative risk in patients receiving multiple FMTs.

No formal causality-attribution method was applied to individual events. Because an adverse event occurring after the procedure cannot reliably be attributed to the FMT itself rather than to bowel preparation, colonoscopy, or anesthesia, all events arising within the 72 h window were counted as procedure-associated regardless of presumed cause. This conservative approach may over-attribute events to FMT but minimizes the risk of underestimating adverse events.

### 2.4. Statistical Analysis

Statistical analyses were performed using IBM SPSS Statistics, version 29 (IBM Corp., Armonk, NY, USA). Median and interquartile range (IQR) were used to describe continuous variables and counts with percentages to describe categorical variables.

Associations between patient and procedural characteristics (sex, age, and underlying disease) and the occurrence of AE were assessed on a per-procedure basis. Because of the small sample and sparse subgroup cells, between-group comparisons of categorical variables relied on Fisher’s exact test; for each disease subgroup versus all others, we derived odds ratios (OR) with 95% confidence intervals (CI). Age, treated as a continuous variable, was compared between procedures with and without AE using the Mann–Whitney U test. A two-sided *p*-value < 0.05 was considered statistically significant.

Given the small number of events (n = 11) and the sparse cell counts within subgroups, we did not fit a multivariable logistic-regression model, since the available data fell short of the number of events per predictor needed for stable estimation. Findings are therefore reported as unadjusted associations only and should be read as preliminary and hypothesis-forming rather than confirmatory. Considering the marked imbalance in subgroup sizes (several indications comprising as few as three procedures) and the multiple comparisons performed without formal correction, the subgroup analyses are severely underpowered and prone to both Type I and Type II error and cannot support conclusions about subgroup-specific risk. Accordingly, the only indication comparison treated as interpretable was ASD versus all non-ASD indications combined. Additionally, to assess the potential impact of repeated procedures within patients, a per-patient sensitivity analysis was performed, classifying a patient as having an AE if any of their procedures was associated with one.

Figure 1 and Figure 2 were created as original vector graphics using Inkscape version 1.4.3.

## 3. Results

### 3.1. Patient Characteristics

A total of 108 FMT procedures were performed on 73 children aged 2 to 17 years. Nineteen patients received more than one FMT; as previously described, each procedure was analyzed as a separate event. The characteristics of patients per procedure are summarized in Table 1.

According to the most recent systematic review of FMT in children, the main indication for FMT in pediatric patients is recurrent rCDI [18]. In contrast, the predominant indication in our cohort was ASD and other developmental disorders (80.6%), reflecting the fact that most patients (or their caregivers) actively sought FMT as a novel treatment option.

### 3.2. Adverse Events

AE were reported after 11 of 108 procedures (10.2%). All AEs were graded as CTCAE grade 1 or 2; no serious AE (CTCAE grade ≥ 3) occurred. The most frequently reported AE was diarrhoea (5.6%) with the duration of 1 to 2 days. The incidence of each AE is detailed in Table 2. Each of the 11 AE-associated procedures occurred in a different child. No patient experienced an adverse event after more than one procedure. Thus, although 19 patients underwent multiple FMTs, AEs were not clustered within individuals, minimizing the statistical dependence that repeated within-patient observations could introduce. On a per-patient basis, 11 of 73 patients (15.1%) experienced at least one AE, similar to the per-procedure rate of 10.2%.

Two cases warrant additional clinical context. One patient (0.9%) who reported a headache following FMT had a two-year history of recurrent headaches, occurring 2–3 times per month and managed with non-steroidal anti-inflammatory drugs. The post-FMT headache was described as more severe than her typical episodes. A second patient (0.9%) developed fever and pharyngitis three days after the procedure; given the timing and clinical presentation, this was considered most likely to represent a community-acquired infection unrelated to the FMT.

### 3.3. Factors Associated with Adverse Events

Associations between patient sex, age, and underlying disease and the occurrence of AEs were assessed (Table 3). No association was observed between sex and AE occurrence (*p* = 1.000). Patient age did not differ between procedures with and without AEs (median [IQR]: AE group 7.7 [5.2–12.8] years vs. no-AE group 7.6 [5.5–12.0] years; Mann–Whitney U = 547.5, *p* = 0.887). When stratified by age group, AEs occurred in 3 of 33 procedures (9.1%) in children aged 2–5 years, 4 of 47 (8.5%) aged 6–11 years, and 4 of 28 (14.3%) aged 12–17 years, with no clear trend across age groups (Table 4). Given the small number of events (n = 11), these age-stratified figures are descriptive.

Because the individual non-ASD indications each comprised as few as three procedures, subgroup-by-subgroup comparison was not statistically meaningful; the only sufficiently powered indication comparison was ASD versus all non-ASD indications combined. Procedures performed for ASD and other developmental disorders were associated with a lower AE rate than non-ASD indications combined (6.9% vs. 23.8%; OR 0.24, 95% CI 0.06–0.87; *p* = 0.036). For completeness, AEs within the non-ASD group occurred in 0 of 3 IBD, 2 of 3 rCDI, 2 of 12 combined IBD and rCDI, and 1 of 3 other procedures; given these very small counts, individual indications were not tested separately and no inference should be drawn from them.

These subgroup analyses are exploratory only and substantially underpowered. Given the marked imbalance in subgroup sizes, the small number of events (n = 11), and the multiple comparisons involved, they are prone to both Type I and Type II error and should be regarded as descriptive observations rather than evidence of subgroup-specific risk.

## 4. Discussion

In this single-centre retrospective study of 108 FMT procedures on 73 children, the overall short-term adverse event (AE) rate was 10.2%, and all AEs were classified as CTCAE grade 1 or 2. No serious AE occurred. A 2022 systematic review and meta-analysis by Wang et al., which included 18 studies and 681 children over 20 years of pediatric FMT experience, reported an overall AE rate of 28.86% (95% CI 19.56–39.15%), with mild-to-moderate events accounting for 27.72% and severe events for only 0.90% [30]. Our rate is well below this pooled estimate and is comparable to two of the largest single-centre pediatric cohorts published to date: Xiao et al. reported a 5.8% AE rate within 48 h among 813 children over 10 years [31], while Zou et al. reported 13.7% per-procedure and 35.1% per-patient rates among 74 children undergoing 508 FMT procedures [32]. The complete absence of CTCAE grade ≥ 3 events in our cohort is consistent with these large-scale series, which have similarly reported very low severe AE rates of 0.2–0.9%.

The most common AEs in our study were diarrhoea (5.6%) and vomiting (2.8%), with isolated reports of headache and fever. It is similar to those described in the pediatric FMT literature, in which gastrointestinal symptoms (abdominal pain, diarrhoea, vomiting) consistently emerge as the most frequent procedure-related events [18,30,31,32]. These symptoms most plausibly reflect transient mucosal and microbial perturbation following the introduction of donor microbiota.

Subgroup analyses identified disease-related differences in AE occurrence. Procedures performed for ASD were associated with a lower AE rate compared with all other indications combined (6.9% vs. 23.8%; OR 0.24, 95% CI 0.06–0.87). Although statistically significant, this association should not be interpreted as a biologically protective effect of the ASD diagnosis itself. It probably reflects the underlying gastrointestinal health of this subgroup relative to the comparator. Patients undergoing FMT for ASD generally lacked active mucosal inflammation, ongoing infection, or other gastrointestinal pathology, in contrast to patients with rCDI, IBD, or combined IBD and rCDI, whose gut environments were affected by active disease at the time of the procedure. This absence of active mucosal disease is the most plausible explanation for the lower adverse event rate in the ASD subgroup, as an inflamed or infected mucosa is more likely to react to bowel preparation, colonoscopy, and the introduction of donor microbiota. This interpretation is supported by direct comparison with published ASD-specific cohorts: in a prospective study of 98 ASD children undergoing FMT, Li et al. reported AE rates of 8.2–10.2% depending on delivery route [33], comparable to the 6.9% we observed in our ASD subgroup. A 2025 systematic review of FMT in pediatric ASD similarly concluded that the procedure is generally safe in this population, with no serious AEs reported across nine studies [34]. Because the overall 10.2% rate is dominated by procedures performed in children without active mucosal inflammation, it should not be extrapolated to settings where FMT is used predominantly for rCDI or IBD, where higher procedural risk may be expected.

Conversely, procedures performed for rCDI showed a numerically higher AE rate. However, this estimate is based on only three rCDI procedures, which is too small for statistical interpretation. Importantly, this finding is not supported by the largest published pediatric series: Xiao et al. did not identify rCDI as an independent risk factor (OR 0.5, *p* = 0.75) [31]. For IBD, our subgroups were not significant (IBD 0%; IBD + rCDI 16.7%), whereas larger series have reported elevated risk—Xiao et al. for IBD (OR 3.98, 95% CI 1.78–8.92) [31], Zou et al. for ulcerative colitis (OR 7.68) [32], and a 2023 systematic review pooling AE and serious AE rates of 29% and 10% [35]. All of our subgroup estimates derive from three to twelve procedures and must be read as descriptive rather than confirmatory; they cannot exclude true differences in risk.

These results support a view of pediatric FMT safety in which the procedure itself is well tolerated, but in which baseline mucosal status and underlying disease activity meaningfully influence the likelihood of transient post-procedural symptoms [17,18,36]. In particular, bowel preparation and colonoscopy frequently produce transient diarrhoea and abdominal discomfort, and general anesthesia can contribute to post-procedural vomiting and headache. Some of the events we recorded (especially diarrhoea, vomiting, and headache) may reflect these peri-procedural factors rather than the transplanted microbiota. Because we counted all events within the window regardless of cause, our adverse event rate conservatively encompasses both procedure- and FMT-related events.

The choice of delivery route is another factor known to influence the AE profile of FMT. Xiao et al. reported AE rates ranging from 2.5% for capsule delivery to 16.0% for nasogastric tube administration, with colonoscopy yielding a 10.3% rate [31] which is very similar to the 10.2% observed in our exclusively colonoscopy-delivered cohort. Some AEs attributed to colonoscopic FMT may reflect peri-procedural factors (bowel preparation, general anesthesia, or the colonoscopy itself) and not the microbiota transplant itself. Wang et al.’s meta-analysis found no significant difference in pediatric AE rates between upper and lower gastrointestinal routes, though comparative pediatric data remain limited [18,30].

### Limitations

Several limitations should be acknowledged. First, the retrospective single-centre design has inherent constraints on data completeness and generalizability, and AEs identified through chart review may be subject to documentation bias.

Second, post-discharge follow-up was passive and relied on families spontaneously contacting the physician: families were provided with direct contact information of the treating gastroenterologist but no scheduled follow-up was performed, which may have led to under-capture of mild self-limiting AEs that resolved without prompting families to contact the team. The true incidence of minor AEs is therefore likely underestimated, and the observed 10.2% rate should be regarded as a lower bound for short-term events.

Third, the cohort was heterogeneous in terms of underlying indication. With more than 80% of procedures performed for ASD and related developmental disorders, which represents a case mix markedly different from the rCDI-predominant populations described in current guidelines, the generalizability of these findings to other pediatric FMT indications is limited. This pattern is increasingly common in some pediatric FMT cohorts, for example, ASD comprised 49.6% of Xiao et al.’s 813-patient series [31]. Nonetheless, the predominance of ASD in our cohort limits direct comparisons across indications and may influence AE reporting patterns, as caregiver expectations and motivation to seek treatment differ across diagnoses.

In addition, the specific donor used for each procedure was not captured in our dataset; consequently, we could not evaluate whether adverse event occurrence varied by donor. Although all donors underwent identical screening and re-screening, a potential donor effect cannot be excluded and warrants assessment in future prospective studies.

With respect to the analysis, although AEs were analyzed primarily per procedure, no patient experienced more than one AE, so the within-patient correlation that could undermine this approach was absent. The higher per-patient rate (15.1% vs. 10.2%) reflects only the smaller patient denominator rather than any clustering of events within individuals, and the overall finding of a low rate of exclusively mild AEs remains unchanged.

Engraftment was not evaluated and could not be assessed retrospectively: microbiome sequencing and donor–recipient strain analyses were not part of clinical care, and no banked samples exist for this cohort. We therefore cannot report whether the transferred microbiota established or persisted in recipients. This reflects both the retrospective design and the study’s focus on procedural safety, and represents an important question for future prospective, microbiome-based studies.

Beyond its clinical findings, this study was conducted with careful attention to ethical rigour: because FMT remains investigational for most of the indications represented here, every procedure was undertaken as individualized clinical care, decided case by case by a multidisciplinary consilium, with families explicitly informed of its investigational status and free to decline without any effect on their child’s continued care. The retrospective analysis of these records was subsequently approved by the regional bioethics committee.

Finally, the AE assessment window was limited to 72 h, and longer-term outcomes, including delayed AEs, were not assessed.

## 5. Conclusions

Within these limitations, our findings support the short-term safety of colonoscopy-delivered FMT in a pediatric population with a heterogeneous mix of indications, with an overall AE rate (10.2%) and severity profile (all CTCAE grade 1–2) that compares favorably with other pediatric FMT literature. Accordingly, these findings should be interpreted as evidence of immediate procedural safety rather than as an assessment of the long-term or overall safety profile of FMT, which will require dedicated longer-term surveillance. Additionally, the findings apply most directly to an ASD-predominant cohort. Larger prospective multicentre studies with longer follow-up and standardized AE reporting are needed to confirm subgroup risk patterns.

## Figures and Tables

**Table 1:** Demographic characteristics of patients per procedure (N = 108).

Characteristic	n (%) or Median [IQR]
Sex	
Male	78 (72.2)
Female	30 (27.8)
Age (years)	7.7 [5.5–12.3]
Indication	
ASD and other developmental disorders	87 (80.6)
Inflammatory bowel disease (IBD)	3 (2.8)
rCDI	3 (2.8)
IBD + rCDI	12 (11.1)
Other ^1^	3 (2.8)

^1^ Includes primary sclerosing cholangitis and two patients with anorexia nervosa. Abbreviations: ASD, autism spectrum disorder; IBD, inflammatory bowel disease; IQR, interquartile range; rCDI, recurrent *Clostridioides difficile* infection.

**Table 2:** AE reported following FMT (N = 108 procedures).

Adverse Event	n (%)
Diarrhoea	6 (5.6%)
Vomiting	3 (2.8%)
Headache	1 (0.9%)
Fever	1 (0.9%)
Total	11 (10.2%)

**Table 3:** Patient characteristics and adverse event occurrence per procedure (N = 108).

Characteristic	Subgroup	No AE, n (%)	AE, n (%)	*p*-Value ^1^	OR ^2^	95% CI
Sex	Male	70 (89.7)	8 (10.3)	1.000	1.03	0.24–4.35
	Female	27 (90.0)	3 (10.0)			
Age, years ^3^	—	7.6 [5.5–12.0]	7.7 [5.2–12.8]	0.887	—	—
Indication	ASD and developmental disorders	81 (93.1)	6 (6.9)	0.036	0.24	0.06–0.87
	Non-ASD indications combined ^4^	16 (76.2)	5 (23.8)			

^1^ *p*-values calculated using Fisher’s exact test for associations between categorical variables and Mann–Whitney U test for age comparisons between groups. ^2^ For the indication comparison, the odds ratio compares ASD with all non-ASD indications combined. ^3^ Age values are median [IQR]; Mann–Whitney U = 547.5. ^4^ Non-ASD indications combined comprises IBD (n = 3), rCDI (n = 3), combined IBD and rCDI (n = 12), and other indications (n = 3); these were pooled because individual subgroups were too small for meaningful comparison. Abbreviations: AE, adverse event; ASD, autism spectrum disorder; CI, confidence interval; IBD, inflammatory bowel disease; IQR, interquartile range; OR, odds ratio; rCDI, recurrent *Clostridioides difficile* infection.

**Table 4:** Adverse event incidence by age group (N = 108 procedures).

Age Group (Years) *	Procedures, n	AE, n	AE Rate, %
2–5	33	3	9.1
6–11	47	4	8.5
12–17	28	4	14.3
Total	108	11	10.2

* Age groups correspond to conventional pediatric developmental stages (preschool, 2–5 years; school-age, 6–11 years; adolescent, 12–17 years); bands were chosen to retain a sufficient number of procedures per group given the small number of events.

## Data Availability

The data presented in this study are available on request from the corresponding author. The data are not publicly available due to privacy and ethical restrictions concerning pediatric patient records.

## Abbreviations

The following abbreviations are used in this manuscript:

AE	adverse event
ASD	autism spectrum disorder
CDI	*Clostridioides difficile* infection
CI	confidence interval
CRP	C-reactive protein
CTCAE	Common Terminology Criteria for Adverse Events
ESPGHAN	European Society for Pediatric Gastroenterology, Hepatology, and Nutrition
ESR	erythrocyte sedimentation rate
FMT	fecal microbiota transplantation
IBD	inflammatory bowel disease
IBS	irritable bowel syndrome
IQR	interquartile range
MRSA	methicillin-resistant *Staphylococcus aureus*
NASPGHAN	North American Society for Pediatric Gastroenterology, Hepatology, and Nutrition
OR	odds ratio
PCR	polymerase chain reaction
rCDI	recurrent *Clostridioides difficile* infection
RPR	rapid plasma reagin
VRE	vancomycin-resistant *Enterococcus*
